# Relationship between monocyte‐platelet aggregation and endothelial function in middle‐aged and elderly adults

**DOI:** 10.14814/phy2.13189

**Published:** 2017-05-22

**Authors:** Andrew Haynes, Matthew D. Linden, Elisa Robey, Louise H. Naylor, Kay L. Cox, Nicola T. Lautenschlager, Daniel J. Green

**Affiliations:** ^1^ School of Sport Science Exercise and Health University of Western Australia Crawley Western Australia Australia; ^2^ School of Pathology and Laboratory Medicine University of Western Australia Crawley Western Australia; ^3^ School of Medicine and Pharmacology (Royal Perth Hospital Unit) University of Western Australia Crawley Western Australia Australia; ^4^ Academic Unit for Psychiatry of Old Age Department of Psychiatry University of Melbourne Melbourne Victoria Australia; ^5^ NorthWestern Mental Health Melbourne Health Parkville Victoria Australia; ^6^ School of Clinical Neurosciences and the Western Australia Centre for Health and Ageing University of Western Australia Crawley Western Australia Australia; ^7^ Research Institute for Sport and Exercise Science Liverpool John Moores University Liverpool United Kingdom; ^8^ Principal Research Fellow National Health and Medical Research Council Canberra Australian Capital Territory Australia

**Keywords:** Cardiovascular disease, endothelial function, platelet function

## Abstract

Low‐grade inflammation, endothelial dysfunction, and platelet hyper‐reactivity to agonists are associated with an increased risk of cardiovascular events. In vitro and animal studies infer an inverse mechanistic relationship between platelet activation and the production of endothelium‐derived nitric oxide and prostacyclin. This concept is supported by evidence of an inverse relationship between endothelial function and platelet activation in high‐risk cardiac patients. The aim of this study was to investigate what relationship, if any, exists between platelet and endothelial function in healthy, middle‐aged, and elderly adults. In 51 participants (18 male, 33 post menopausal female), endothelial function was assessed by flow‐mediated dilation (FMD). Platelet function was assessed by flow cytometric determination of glycoprotein IIb/IIIa activation (measured by PAC‐1 binding), granule exocytosis (measured by surface P‐selectin expression), and monocyte‐platelet aggregates (MPAs), with and without stimulation by canonical platelet agonists adenosine diphosphate (ADP), arachidonic acid (AA), and collagen. Correlation analysis indicated there was no significant (all *P *=> 0.05) relationship between FMD and any marker of in vivo platelet activation (MPAs *R *=* *0.193, PAC‐1 *R *= −0.113, anti‐CD62P *R *= −0.078) or inducible platelet activation by ADP (MPA *R *= −0.128, anti‐CD62P *R *= −0.237), AA (MPA *R *= −0.122, PAC‐1 *R *= −0.045, anti‐CD62P *R *= −0.142), or collagen (MPA *R *=* *0.136, PAC‐1 *R *=* *0.174, anti‐CD62P *R *= −0.077). Our findings contrast with two previous studies performed in high‐risk cardiac patients, which reported inverse relationships between platelet activation and endothelial function, suggesting that some compensatory redundancy may exist in the relationship between platelet and endothelial function in preclinical populations.

## Introduction

Thrombosis is an integral component of acute coronary syndromes such as acute myocardial infarction (MI) and ischemic stroke (Falk et al. 2013; Libby 2013). Moreover, the role of platelets in the early, dormant stages of atherosclerosis and cardiovascular disease (CVD) has recently been recognized (Lievens and von Hundelshausen 2011; Rondina et al. 2013). Low‐grade inflammation, endothelial dysfunction, and platelet hyper‐reactivity to agonists are all independently associated with an increased risk of cardiovascular events (Gurbel et al. 2007; Libby et al. 2009). An intact and healthy endothelium regulates and inhibits platelet activation (Davì and Patrono 2007) and endothelial dysfunction may play a direct role in activating platelets (Davì et al. 2002). Platelet activation results in the formation of monocyte‐platelet aggregates (MPAs) (Linden 2013), and stepwise increases in MPAs and activated platelets have been observed with cardiovascular disease progression (Linden et al. 2007). The interaction between activated platelets and monocytes can initiate the release of proinflammatory and adhesive molecules that are atherogenic (Michelson et al. 2001; McFadyen and Kaplan 2015; Yuan et al. 2015; Gerdes et al. 2016), driving a proatherogenic monocytic phenotype (Barnard et al. 2005) and facilitating the infiltration of monocytes to the subintima space (Weyrich et al. 1996; McFadyen and Kaplan 2015).

A number of in vitro experiments suggest that nitric oxide (NO) and prostacyclin (PGI2), produced by endothelial cells, directly inhibit platelet aggregation (Alheid et al. 1987, 1989; Macdonald et al. 1988). However, the functional relationship between platelet activation and endothelial function in vivo is less clear (Radomski et al. 1987; Megson et al. 2000). While inhibition of NO production in healthy young adults can increase platelet activation (Schäfer et al. 2004b) and decrease clotting time (Simon et al. 1995), this is not a universal finding (Albert et al. 1999) and these studies assessed the acute impact of pharmacological blockade. An inverse relationship between endothelium‐dependent coronary vasomotor function and MPAs measured in arterial samples has been documented in high‐risk patients undergoing cardiac angiography and angioplasty (Hamilos et al. 2011; Di Serafino et al. 2014). There is also evidence to suggest that pharmacological blockade of the platelet fibrinogen receptor (glycoprotein IIb/IIIa receptor) (Heitzer et al. 2003) and administration of the antiplatelet medications clopidogrel (Hamilos et al. 2008; Warnholtz et al. 2008; Muller et al. 2010; Patti et al. 2011) and aspirin (Husain et al. 1998; Williams et al. 2005) can acutely improve endothelial function in patients with CAD. However, these short‐term effects were abolished when administration was maintained for longer periods of time (Ostad et al. 2011). One previous study (Lanza et al. 2011) has investigated the relationship between in vivo endothelial function, assessed using brachial artery flow‐mediated dilation (FMD) and platelet function assessed via flow cytometry. This study was conducted in adolescents, with or without a positive family history of CAD, and no relationship was found between FMD and platelet activation (MPAs, PAC‐1 or CD62P binding).

Increasing age and physical inactivity are risk factors for cardiovascular disease (Sesso et al. 2000), but no previous study, to our knowledge, has investigated relationships between endothelial function and platelet activation in asymptomatic healthy older adults, or whether any such relationship extends to agonist‐induced platelet activation. Therefore, our aim was to test the relationship, if any, between platelet and endothelial function in apparently healthy, physically inactive, middle‐aged, and elderly humans. We hypothesized that FMD would be inversely associated with the presence of MPAs and activated platelets; and inversely associated with agonist‐induced platelet activation.

## Materials and Methods

The study was approved by the University of Western Australia Human Research Ethics Committee, procedures were in accord with the Declaration of Helsinki and participants provided written informed consent.

Male and postmenopausal female participants were recruited from the general population in Perth, Western Australia using multiple recruitment strategies including advertisements in local newspapers, radio stations, and posters. Apparently healthy individuals aged 45 years and over were encouraged to contact the research team, resulting in initial phone screening procedures which included questionnaires to determine suitability to attend a formal screening visit. Initial exclusion criteria included serious illness such as cancer, diagnosed cognitive impairment or dementia, current or past history of ischemic heart disease, angina, stroke, persistent arrhythmias, diabetes mellitus, airway disease, epilepsy, severe mental illness, engaging in more than 1 h of physical activity per week, current or recent smokers (within 12 months), pre‐ or peri‐menopausal females and alcohol consumption >28 standard drinks/wk.

Individuals satisfying the initial criteria were invited to attend a screening session during which a number of measures were collected including: height, body mass, resting electrocardiogram (ECG), and fasting blood tests (glucose, lipid profile, full blood count, urea, and electrolytes). Participants exhibiting abnormal cardiac rhythms, blood test results suggestive of chronic kidney disease, diabetes, or total cholesterol >7 mmol/L were excluded. Included participants were then invited to perform an exercise stress test with ECG monitoring and those with evidence of exertion‐induced myocardial ischemia were excluded from further participation. In subsequent visits, participants underwent a 20 min resting blood pressure (BP) assessment and a dual‐energy x‐ray absorptiometry (DEXA) scan. For the resting BP assessment, participants arrived at the laboratory in the morning following an overnight fast and lay in a supine position in a temperature‐controlled dark room. BP was measured every 2 min by an automated device (Dinamap V100, GE Healthcare, USA). Individuals with an average systolic BP >160 mmHg or diastolic BP >100 mmHg were excluded.

For FMD and platelet function tests, participants arrived at the laboratory in the morning between 7:00 and 9:30am, following an overnight fast, having abstained from the consumption of caffeine and alcohol for 12 and 24 h, respectively, and not taken part in physical exercise for 24 h. Adherence to the protocol was confirmed by questionnaire on arrival. Prior to attending the laboratory for data collection, participants were instructed to be clear of symptoms for 7 days if they had recently suffered with acute conditions including respiratory tract infection, cold, and flu. Participants taking prescription medications were instructed to maintain their usual routine of administration. However, the use of nonprescribed medications such as anti‐inflammatory, antihistamine, antibiotic, aspirin, cold, and flu medications were ceased for at least 7 days prior to blood collection. Participants lay supine in a cool temperature‐controlled room for 15 min, after which a blood sample was collected from the dominant arm for the assessment of platelet function. Subsequent FMD tests were performed on the nondominant arm.

### Blood collection

A venous blood sample was collected into a 4 mL 3.2% sodium citrate tube (Vacuette by Greiner bio‐one) and processed according to published standards (Linden 2013), as preanalytical variables during blood collection, storage, and handling are an important factor in platelet function testing. Within 10 min of collection, blood was processed to assess circulating levels of MPAs and activated platelets and their sensitivity to platelet agonists.

### Monocyte‐platelet aggregates

Each MPA reaction tube included two antibodies: CD14 (monocyte identifier) conjugated to the fluorophore Brilliant Violet (BV) 421 (Clone M5E2, BioLegend, San Diego CA) and CD42b (platelet identifier) conjugated to Allophycocyanin (APC) (Clone HIP1, BioLegend) or IgG isotype control (BioLegend). Six MPA reaction tubes (Protein LoBind Eppendorf, Germany) were included: isotype control, no agonist, positive control (250 *μ*mol/L thrombin receptor activating peptide‐6 (SFLLRN, Sigma‐Aldrich, MO)), and threshold (low) concentrations of the following three agonists: adenosine diphosphate (ADP) 1.5 *μ*mol/L (Chrono‐Log Corp., PA), arachidonic acid (AA) 10 *μ*g/mL (Sodium arachidonate, Bio/Data Corp., PA), and collagen 1.5 *μ*g/mL (Chrono‐Log Corp., PA). Absence of spectral overlap was confirmed by single‐color comp bead controls (BD Biosciences). Samples were fixed and red cells lysed with 800 *μ*L of BD FACSLyse solution (BD Biosciences) following exactly 15 min of incubation. Samples were then stored in the dark at 4°C and analyzed by flow cytometry (BD FACSCanto^™^ II, BD Biosciences) at a low flow rate for 10 min per tube, to avoid coincident events (Hui et al. 2015).

### Platelet surface receptors

Whole blood was diluted 1:5 with HEPES saline buffer and incubated with a cocktail of the following fluorescent conjugated antibodies at saturating concentrations: PAC‐1 fluorescein (FITC), CD62P phycoerythrin (PE), CD42b PE‐Cy5, or IgG1Κ PE isotype control (all BD Pharmingen) for exactly 15 min. Six reaction tubes were used that were identical in function and agonist concentrations as those used for MPAs. However, ADP at the concentration used (1.5 *μ*mol/L) caused maximal PAC‐1 binding in all participants, so was not included in statistical analysis. Following incubation, samples were fixed with 800 *μ*L of stabilizing fixative (BD Biosciences) and were then stored at 4°C until analysis by flow cytometry (BD FACSCanto II) within 24 h. Samples were run at a low flow rate until 10,000 platelet positive events were counted. To account for spectral overlap between the three fluorophores, single‐stained compensation beads were used (BD Biosciences). For both MPAs and platelet surface receptor binding, samples were incubated at room temperature with the exception of tubes containing AA and collagen, which were incubated at 37°C using a dry block heater (Ratek DBH20D, Victoria, Australia).

### Flow‐mediated dilation

The vascular assessments were conducted in a quiet, temperature‐controlled room in accordance to recent guidelines (Thijssen et al. 2011). To examine brachial artery FMD, the nondominant arm was extended and positioned at an angle of ~80° from the torso. A rapid inflation and deflation pneumatic cuff (D.E. Hokanson, Bellevue, WA) was positioned on the forearm, immediately distal to the olecranon process to provide a forearm ischemia stimulus. Using this approach, the brachial artery dilation represents a largely NO‐mediated, endothelium‐dependent response (Green et al. 2011). A 10‐MHz multifrequency linear array probe, attached to a high‐resolution ultrasound machine (T3200; Terason, Burlington, MA) was used to image the brachial artery in the distal 1/3rd of the upper arm. When an optimal image was obtained, the probe was held stable and the ultrasound parameters were set to optimize the longitudinal, B‐mode images of lumen–arterial wall interface. Continuous Doppler velocity assessments were also obtained using the ultrasound, and were collected using the lowest possible insonation angle (always <60°). Following a 1‐min baseline recording of brachial artery diameter and velocity (Camtasia Studio 8, TechSmith, Okemos, MI), which were used to examine baseline blood flow patterns, the forearm cuff was inflated (220 mmHg) for 5 min. Diameter and flow recordings resumed 30s prior to cuff deflation and continued for 3 min thereafter. Post‐test analysis of brachial artery diameter was performed using custom‐designed edge‐detection and wall‐tracking software, which is largely independent of investigator bias (Woodman et al. 2001). Brachial artery FMD is presented as relative (%) rise from the preceding baseline diameter. We have shown that the reproducibility of diameter measurements using this semiautomated software is significantly better than manual methods, reduces observer error significantly, and possesses an intraobserver CV of 6.7%.

### Statistical methods

To test whether there was a relationship between FMD and platelet function, a Pearson product‐moment correlation test was carried out for data that were normally distributed. For data that were not normally distributed, a Spearman's Rank test was conducted. Subsequently, to determine if potential confounding factors including: age, gender, resting heart rate and blood pressure, body fat percentage, fasting blood glucose and lipids, and medication use impacted upon the results, partial correlations were conducted to account for these covariates. Participants were then divided into two groups based on gender, and correlation analysis between FMD and platelet function was conducted for males and females separately to determine if results were gender specific. To test whether any differences existed between male and female participants for FMD or any of the platelet function variables measured, independent sample T‐tests and Mann–Whitney U‐tests were conducted for data meeting and failing normality assumptions, respectively.

## Results

Fifty‐one participants (18 male, 33 female) were included in the study and underwent platelet function and FMD tests. The general characteristics of participants included in the study are presented in Table 1. Two participants failed to receive a DEXA scan, so for data derived using this outcome measure *n* = 49. Descriptive statistics for vascular and platelet function outcome measures can be seen in Table 2. An error in the processing of platelet PAC‐1 and CD62P with no agonist occurred for 1 participant and for PAC‐1 and CD62P with AA incubation for 1 other participant. Therefore, for the direct comparison of all MPA data vs. FMD *n* = 51, but for PAC‐1 and CD62P NA and AA *n* = 50.

**Table 1:** General characteristics, anthropometric, and dual‐energy x‐ray absorptiometry (DEXA) and biochemistry variables

	All participants	Male	Female
Age (years)	60.6 ± 7.4	57.1 ± 6.3	62.3 ± 7.6
Anthropometric data			
Height (cm)	166.5 ± 8.0	173.8 ± 4.8	162.2 ± 6.0
Body mass (kg)	76.9 ± 15.8	89.8 ± 11.1	70.0 ± 13.0
Body mass index (kg/m^2^)	27.6 ± 4.5	29.8 ± 3.7	26.6 ± 4.6
Total body fat % (DEXA) *n* = 49	39.5 ± 7.3	34.1 ± 1.2	42.4 ± 6.8
Resting HR & blood pressure			
Heart rate (bpm)	62 ± 7		
Systolic BP (mmHg)	123 ± 14	127 ± 12	121 ± 14
Diastolic BP (mmHg)	72 ± 9	79 ± 9	69 ± 6
Mean arterial pressure (mmHg)	92 ± 10	98 ± 9	89 ± 8
Fasting Biochemistry (mmol/L)			
Cholesterol	5.6 ± 0.9	5.5 ± 1.1	5.6 ± 0.7
Triglyceride	1.2 ± 0.7	1.6 ± 0.6	1.1 ± 0.7
LDL‐C	3.6 ± 0.8	3.6 ± 0.9	3.6 ± 0.7
HDL‐C	1.4 ± 0.3	1.1 ± 0.2	1.5 ± 0.3
Glucose	5.1 ± 0.4	5.3 ± 0.1	5.0 ± 0.1
Prescription Medication	*N* (dual meds)		
Any medication	12 (3)	3	9
Blood Pressure medication total	6 (3)	2	4 (3)
*Ca channel block*	2		
*Beta Blocker*	1		
*Angiotensin II receptor antagonist*	3		
Statins	6 (3)	1	5 (3)
Antidepressant	3		3

Dual‐energy X‐ray absorptiometry *DEXA*, Heart rate *HR*, Blood pressure *BP*, Low‐density lipoprotein cholesterol *LDL‐C*, High‐density lipoprotein cholesterol *HDL‐C*. Values are Mean ± SD with exception of Prescription Medication presented as total N. *N* = 51 unless stated otherwise.

**Table 2:** Descriptive statistics of FMD and platelet function tests, and results of correlation tests between FMD% and platelet function

	Mean ± SD	*R*	*P*
FMD%	4.6 ± 2.5		
BD (mm)	3.6 ± 0.7		
PD (mm)	3.8 ± 0.7		
Platelet Variable %			
MPA NA	4.1 ± 1.4	0.193	0.175
MPA ADP1.5 *μ*mol/L	47.8 ± 16.8	−0.128	0.369
MPA AA10 *μ*g/mL	20.8 ± 25.3	−0.122	0.396
MPA Coll 1.5 *μ*g/mL	5.3 ± 5.5	0.136	0.34
PAC‐1 NA (*n* = 50)	4.7 ± 5.4	−0.113	0.433
PAC‐1 AA10 *μ*g/mL (*n* = 50)	23.3 ± 17.3	−0.045	0.755
PAC‐1 Coll 1.5 *μ*g/mL	22.3 ± 20.6	0.174	0.223
CD62P NA (*n* = 50)	1.8 ± 1.4	−0.078	0.591
CD62P ADP1.5 *μ*mol/L	58.7 ± 19.1	−0.237	0.094
CD62P AA10 *μ*g/mL (*n* = 50)	13.5 ± 11.8	−0.142	0.324
CD62P Coll 1.5 *μ*g/mL	8.8 ± 10.1	−0.077	0.591

FMD, *Flow‐mediated dilation,* BD, *baseline diameter,* PD, *peak diameter,* Monocyte‐platelet aggregate *MPA*, No agonist *NA*, Adenosine diphosphate *ADP*, Arachidonic acid *AA*, Collagen *Coll. N* = 51 unless stated otherwise.

### Relationships between FMD and platelet activation

**Figure 1:**
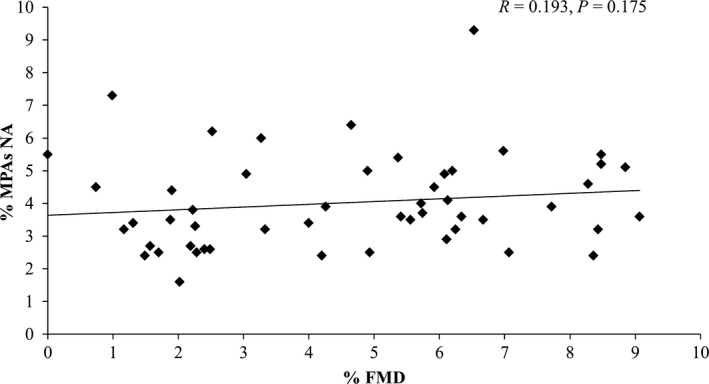
Individual responses for MPAs no agonist (NA) versus flow‐mediated dilation (FMD%) and results of correlation analysis. *N* = 51.

**Figure 2:**
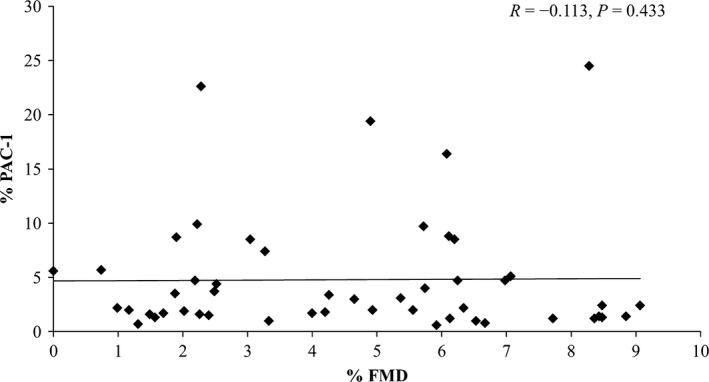
Individual responses for platelet PAC‐1 binding no agonist (NA) versus flow‐mediated dilation (FMD%) and results of correlation analysis. *N* = 50.

**Figure 3:**
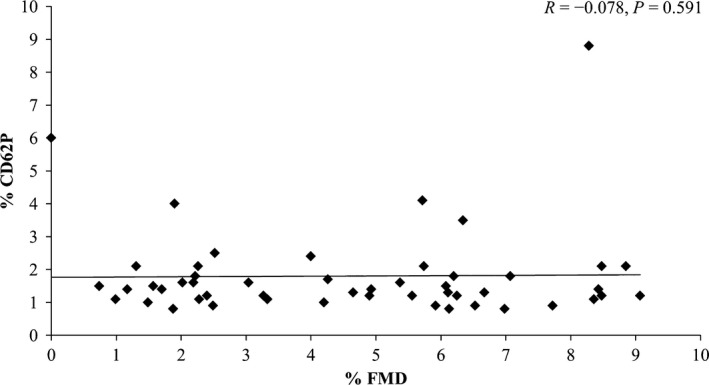
Individual responses for platelet anti‐CD62P binding no agonist (NA) versus flow‐mediated dilation (FMD%) and results of correlation analysis. *N* = 50.

No significant relationship was found between FMD% and circulating levels of activated platelets, whether measured by MPAs (Fig. 1), PAC‐1 (Fig. 2) or anti‐CD62P (Fig. 3) binding (all *P *>* *0.05). No relationship was observed between FMD% and platelet reactivity to the agonists ADP (MPA & CD62P only), AA or collagen for MPAs, PAC‐1 or anti‐CD62P binding (all *P *>* *0.05). All correlation results can be found in Table 2.

### Impact of potential confounding variables

Inspection of scatter plots suggested that no linear relationship existed between any of the potential confounding variables (age, gender, resting heart rate, blood pressure, % body fat, fasting glucose, and lipids (triglycerides, cholesterol, LDL, and HDL) and medication use) and FMD% or any of the platelet function parameters. Consequently, the results of partial correlation testing indicated that no significant relationship (all *P *>* *0.05) existed between FMD% and any of the measures of platelet function we studied when these potentially confounding variables were included as cofactors (see Table 3).

**Table 3:** Results of partial correlation tests between FMD% and platelet function accounting for the potential influence of covariates

Platelet Variable	*R*	*P*
MPA NA	0.119	0.484
MPA ADP1.5 *μ*mol/L	−0.071	0.675
MPA AA10 *μ*g/mL	0.015	0.928
MPA Coll 1.5 *μ*g/mL	−0.091	0.592
PAC‐1 NA (*n* = 50)	−0.075	0.664
PAC‐1 AA10 *μ*g/mL (*n* = 50)	−0.155	0.368
PAC‐1 Coll 1.5 *μ*g/mL	−0.005	0.975
CD62P NA (*n* = 50)	0.066	0.704
CD62P ADP1.5 *μ*mol/L	−0.246	0.143
CD62P AA10 *μ*g/mL (*n* = 50)	−0.091	0.598
CD62P Coll 1.5 *μ*g/mL	0.034	0.844

FMD *Flow‐mediated dilation,* Monocyte‐platelet aggregate *MPA*, No agonist *NA*, Adenosine diphosphate *ADP*, Arachidonic acid *AA*, Collagen *Coll*. Covariates include: age, gender, body fat percentage, resting heart rate and blood pressure, fasting glucose and lipids and medication use. *N* = 51 unless stated otherwise.

### Potential gender differences

Independent correlation analyses conducted for male and female participants individually revealed there were no significant relationships between FMD and any of the platelet function variables we measured for either gender (all *P *= >0.05). No significant differences were found between male and female participants for FMD (*P *=* *0.767), MPA NA (*P *=* *0.554), MPA ADP (*P *=* *0.577), MPA AA (*P *=* *0.419), MPA collagen (*P *=* *0.608), PAC‐1 NA (*P *=* *0.928), PAC‐1 AA (*P *=* *0.524), PAC‐1 collagen (*P *=* *0.150), CD62P NA (*P *=* *0.146), CD62P ADP (*P *=* *0.585), CD62P AA (*P *=* *0.565), CD62P collagen (*P *=* *0.086).

## Discussion

Endothelial dysfunction and increased platelet activation are both associated with cardiovascular disease progression (Davì et al. 2002; Davignon J. 2004) and, based on in vitro and animal studies, it is often inferred that a direct and inverse relationship exists between the function of the endothelium and platelet activation (Alheid et al. 1987, 1989; Schäfer et al. 2004a,b). The aim of this study was to investigate the relationship between endothelial function and platelet activation in a preclinical low‐risk population of older participants with low physical activity levels. In this study, we did not find any relationship between flow‐mediated dilation, an indicator of endogenous nitric oxide and prostacyclin bioavailability (Green et al. 2011), and platelet function assessed by flow cytometry.

Our findings were unexpected, as the extant literature suggests there is an inverse relationship between platelet and endothelial function (Husain et al. 1998; Heitzer et al. 2003; Schäfer et al. 2004a,b; Hamilos et al. 2008, 2011; Warnholtz et al. 2008; Muller et al. 2010; Patti et al. 2011; Di Serafino et al. 2014). In contrast to our study, two previous investigations have reported an inverse relationship between vascular function and MPAs in high‐risk cardiac patients (Hamilos et al. 2008; Di Serafino et al. 2014). These studies utilized highly invasive vascular tests (coronary vasomotor function) and measured platelet activation in arterial blood samples. We tested endothelial function in the brachial artery following established guidelines, a common and reproducible approach (Thijssen et al. 2011; Mosawy et al. 2012). Platelet function was interrogated in venous blood samples with multiple outcomes (i.e., MPAs, PAC‐1 and anti‐CD62P binding), with and without canonical platelet agonists, using sophisticated and established flow cytometry techniques (Linden 2013) which have been associated with cardiovascular outcome. It is possible that differences in participant characteristics and/or methodological approaches between our study and these previous reports are responsible for the divergent findings. In critically ill patients with sepsis, MPAs in arterial blood were ~60% greater compared to MPAs in venous blood (Rondina et al. 2012), but to our knowledge no investigation has determined if sampling technique confers physiological or pathological relevance, or if such a difference exists in healthy individuals.

The majority of studies that have reported an inverse relationship between platelets and endothelial function have involved pharmacological modulation of these variables using acute experiments in humans (Husain et al. 1998; Heitzer et al. 2003; Schäfer et al. 2004b; Hamilos et al. 2008; Warnholtz et al. 2008; Muller et al. 2010; Patti et al. 2011). In one such study, platelet activation was increased by blockade of endothelium‐derived nitric oxide using a bolus dose of N^*G*^‐monomethyl‐L‐arginine (Schäfer et al. 2004b), whereas lower doses of blockade did not induce such changes (Albert et al. 1999). Likewise, increases in FMD have been observed following a single loading dose of clopidogrel and vasodilation responses postadministration were dose‐dependent (Warnholtz et al. 2008). Research in which a significant association was observed between platelet and endothelial function has involved participants with established coronary disease undergoing cardiac angiography and angioplasty. Such individuals exhibit large changes in platelet activation and endothelial function (Linden et al. 2007; Falk et al. 2013) and possess an inflammatory state (Libby et al. 2009) that may partly explain the inverse relationship observed. In contrast, there is some evidence to suggest upregulation of NO‐synthase‐mediated endothelial function occurs in the presence of CVD risk factors (Minor et al. 1990; Cosentino et al. 1997) and it is possible that, in subclinical and apparently healthy populations with risk factors for CVD, some compensatory redundancy exists in the interaction between the endothelium and platelets. This could account for the lack of an inverse relationship between these two variables in our study. Furthermore, there is some evidence to suggest that compensatory mechanisms that prevent spontaneous thrombosis and preserve hemostatic function occur in mice incapable of producing NO (Iafrati et al. 2005). Indeed, the percentage of circulating MPAs in our cohort (4.1 ± 1.4%) were far lower than that reported in samples taken from high‐risk cardiac (~20–38%) (Hamilos et al. 2011; Di Serafino et al. 2014) and stable CAD (~12%) patients (Linden et al. 2007), even after considering the potential differences in MPAs between arterial and venous blood (Rondina et al. 2012).

Our findings do not necessarily conflict with the established role of platelet and endothelial function in atherosclerosis, highlighted in recent reviews (Davì et al. 2002; McFadyen and Kaplan 2015; Cahill and Redmond 2016). However, because atherosclerosis evolves slowly over many decades in humans, it is possible that the relationship between endothelial and platelet function in healthy individuals is less apparent and/or detectable, even with the sensitive technical approaches used in this study. In contrast, transgenic mouse models provide a relatively accelerated and exaggerated insight into disease pathogenesis (Breslow 1996). Diabetic mice with lower NO bioavailability exhibit higher basal levels of activated platelets compared to control mice (Schäfer et al. 2004a), whereas mice with induced diabetes but preserved NO bioavailability due to overexpression of tetrahydrobiopterin possess normal levels of activated platelets. These findings suggest that in accelerated pathological models and/or in certain advanced disease states (e.g., CAD, diabetes mellitus), increased platelet activation and reduced endothelial NO may be mechanistically relevant. Although we deliberately recruited healthy middle‐aged and elderly individuals in this study, future research should include a wide range of participants, across the lifespan. A potential limitation to this study is the limited sample size, but previous studies which have reported a significant inverse relationship between FMD and platelet activation have included 30 (Di Serafino et al. 2014) and 19 (Hamilos et al. 2008) patients, so this is unlikely to be responsible for the lack of relationship in this study.

There are several limitations to our study. We constrained recruitment to >45 years and all women were postmenopausal. Our average FMD results indicate a relatively lower response than studies involving younger cohorts, in keeping with the established impact of age on vascular function. Nonetheless, we cannot generalize our findings to younger or much older age groups. Similarly, our study excluded participants with established cardiovascular diseases and, as mentioned above, correlations between platelet and endothelial function may be more apparent in those with overt disease. Finally, while some of our participants were taking medications, subgroup analysis revealed no impact of drugs on the magnitude or pattern of correlation in our study. Notably, all participants taking medications specifically known to impact upon platelet function were excluded from our study.

In summary, we did not observe any relationship between endothelial function and platelet activation in the healthy, middle‐aged, and elderly participants we recruited in this study. As our findings contrast with some previous studies conducted in high‐risk cardiac patients, our data suggest that some compensatory redundancy may exist in the relationship between platelet and endothelial function in preclinical populations.

## Conflict of Interest

None.
